# An Australian perspective of using video for the assessment of laparoscopic surgery and support for artificial intelligence in performance evaluation

**DOI:** 10.1007/s00423-026-04037-y

**Published:** 2026-03-30

**Authors:** Yuchen Luo, Shekhar Kumta, Wanda Stelmach, Russell Hodgson

**Affiliations:** 1 Division of Surgery, Northern Health, Epping, Australia; 2 Department of Surgery, University of Melbourne, Epping, VIC Australia; 3 Department of Health and Biomedical Sciences, RMIT, Bundoora, VIC Australia

**Keywords:** Surgical training, Surgical education, Laparoscopy, Laparoscopic cholecystectomy, Video recording, Video assessment, Artificial intelligence, Computer vision

## Abstract

**Background:**

The objective evaluation of technical competence in laparoscopic surgery is critical, yet many current assessment tools lack precision and video-based assessment is not routinely integrated into formal training. This survey gauges a national consensus on video-based assessment of cholecystectomy and the perspectives of use of artificial intelligence in the future.

**Methods:**

This cross-sectional survey study utilized a national survey distributed to the Australian general surgery community via the REDCap platform. The survey assessed proposed criteria for the dissection and excision phases of laparoscopic cholecystectomy and explored surgeons’ perspectives on the future use of video recordings and artificial intelligence in surgical training.

**Results:**

With a 20.0% response rate (192/962), the survey revealed a strong consensus among surgeons regarding the proposed assessment criteria, with key items like non-targeted diathermy burning and incorrect clipping achieving high agreement. While video recording for review is not routine, a significant proportion of participants expressed that video-based assessment is “somewhat likely” or “very likely” to become mandatory for competency evaluation. Attitudes toward integrating AI and software tools for video-based assessment were generally positive.

**Conclusion:**

This study demonstrates a clear consensus among general surgeons on objective assessment criteria for laparoscopic cholecystectomy and signals a shift towards formal video-based assessment and AI integration in surgical training. These findings are crucial for developing reliable assessment tools and integrating advanced technologies to enhance surgical education and trainee evaluation.

**Supplementary Information:**

The online version contains supplementary material available at 10.1007/s00423-026-04037-y.

## Introduction

 Over the past decade, the practice of recording laparoscopic surgery for performance evaluation has gained global traction. Current research highlights the development of various assessment tools primarily focused on three aspects: global skills, operative errors and procedure specific performance. However, many of these tools lack precision and detailed definitions for certain assessment criteria [1, 2]. While some surgeons utilize video recordings for self-improvement, video-based assessment is not a fundamental part of formal surgical training programmes. The prevailing General Surgery Education Training Program (GSET) emphasizes the importance of trainer-trainee interaction for feedback on procedural performance and assessment of technical competence in key operations [3]. This raises the question of how supervisors can more objectively evaluate individual trainee performance. Should they rely on some of the existing subjective global rating scales, for example rating “excellent” versus “very bad” in Calot’s triangle dissection, or “no understanding” versus “excellent understanding” of a procedure’s knowledge [4]? Alternatively, should they adopt more quantifiable assessment metrics, such as error frequency, achievement of critical steps, or time taken for specific phases?

The assessment items were selected based on a comprehensive departmental panel discussion amongst hepatopancreatobiliary surgeons, extensive literature review and preliminary explorations via a pilot study conducted at Northern Health, Melbourne, Australia [5]. This cross-sectional survey study was achieved through a national survey distributed to the general surgery community, and it also explored Australian surgeons’ perspectives on utilizing video recordings and artificial intelligence (AI) in future surgical training.

## Methods

A survey was developed using the REDCap platform to evaluate proposed assessment criteria for the dissection and excision phases of laparoscopic cholecystectomy. The survey was disseminated via email to members of the General Surgeons Australia (GSA). It serves as a critical component of a Delphi process to develop an effective, reliable, and valid assessment tool, incorporating diverse expert perspectives to ensure comprehensiveness of the assessment blueprint for laparoscopic cholecystectomy. All collected data were anonymized and secured with an encrypted password. Descriptive analysis was performed using REDCap. Ethical approval was obtained (2025_Non-HREC_10). The development of this survey is demonstrated in accordance with CHERRIES guidelines. (Supplementary 1)

## Results

This survey achieved a response rate of 20.0%, with 192 out of 962 active members of GSA specialist surgeons responded. The majority of respondents indicated agreement with the proposed assessment criteria for both the dissection and excision phases of laparoscopic cholecystectomy. None of the assessment items in either dissection or excision phase were graded by more than 25% of respondents as “not useful”. In the dissection phase, non-targeted diathermy burning, uncontrolled tearing of tissue and incorrect clipping reached significant agreement with approximately 80% of respondents rating these as “very useful”. In the excision phase, criteria such as dissection into liver, non-targeted diathermy burning, incorrect clipping all achieved high agreement among at least 60% of the participants. Key additional feedback highlighted the importance of presence and management of intraoperative bleeding, performance of cholangiocatherisation and achieving critical view of safety. (Table 1)

**Table 1:** Survey results

	Not useful	Somewhat useful	Very useful	
Dissection phase				
No. of times grasping Hartmann’s pouch	32/139 (23.0%)	73/139 (52.5%)	34/139 (24.5%)	
No. of times rotating gallbladder front to back (or vice versa) (up to achieving view of safety)	27/139 (19.4%)	62/139 (46.0%)	48 (34.5%)	
Non-targeted diathermy	10/140 (7.1%)	20/140 (14.3%)	110 (78.6%)	
Uncontrolled tearing of tissue	2/139 (1.4%)	24/139 (17.3%)	113 (81.3%)	
No. of times of instrument clashes	17/140 (12.1%)	63/140 (45.0%)	60/140 (42.9%)	
Incorrect clipping	3/140 (2.1%)	22/140 (15.7%)	115/140 (82.1%)	
Lack of progress for one minute	16/140 (11.4%)	83/140 (59.3%)	41 (29.3%)	
Gallbladder perforation	22/140 (15.7%)	97/140 (69.3%)	21 (15.0%)	
No. of times instrument inserted into the working port	32/140 (22.9%)	76/140 (54.3%)	32 (22.9%)	
Excision phase				
Gallbladder perforation	14/134 (10.4%)	96/134 (71.6%)	24/134 (17.9%)	
Dissection into liver	0	48/134 (35.8%)	86 (64.2%)	
Non-targeted diathermy	5/134 (3.7%)	34/134 (25.4%)	95/134 (70.9%)	
No. of times of instrument inserted into the working port	25/133 (18.8%)	80 (60.2%)	28 (21.1%)	
Incorrect clipping	7/134 (5.2%)	45/134 (34.3%)	79/134 (60.4%)	
Personal perspectives				
Record laparoscopic videos	Yes (35/128, 26%)	No (93/128, 74%)		
Reason for recording videos	Teaching/Training (41/65, 62%)	Self-assessment (35/65, 52%)	Quality assurance (16/65, 25%)	Medico-legal (15/65, 25%)

**Fig. 1:**
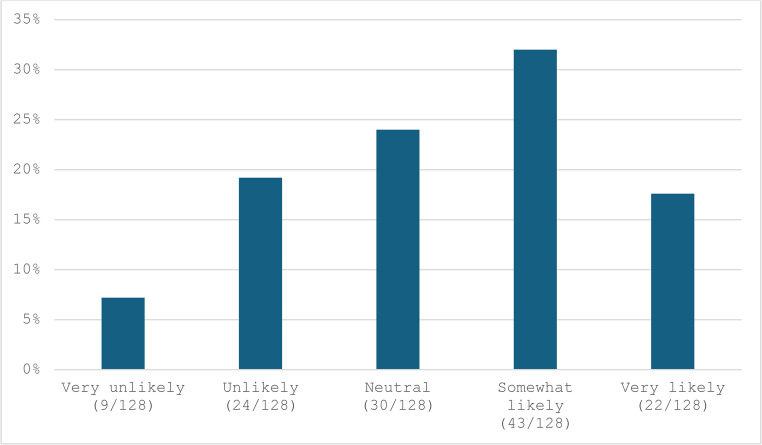
How likely do you think video-based assessment of laparoscopic surgery becomes mandatory part of formal surgical training for assessing procedural competency?

**Fig. 2:**
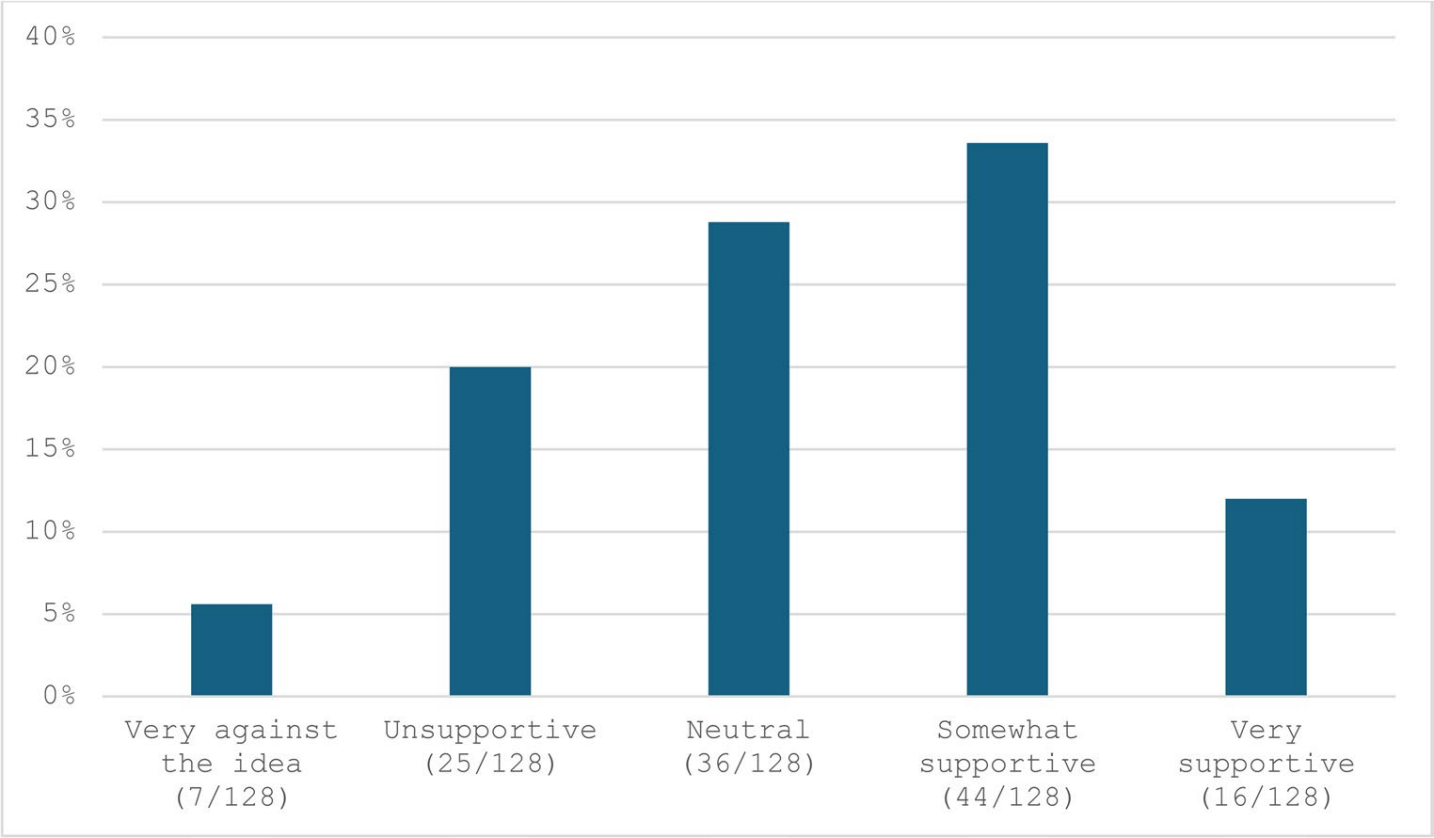
How do you feel about using artificial intelligence (AI) or other software tools for video-assessment of laparoscopic surgery?

**Fig. 3:**
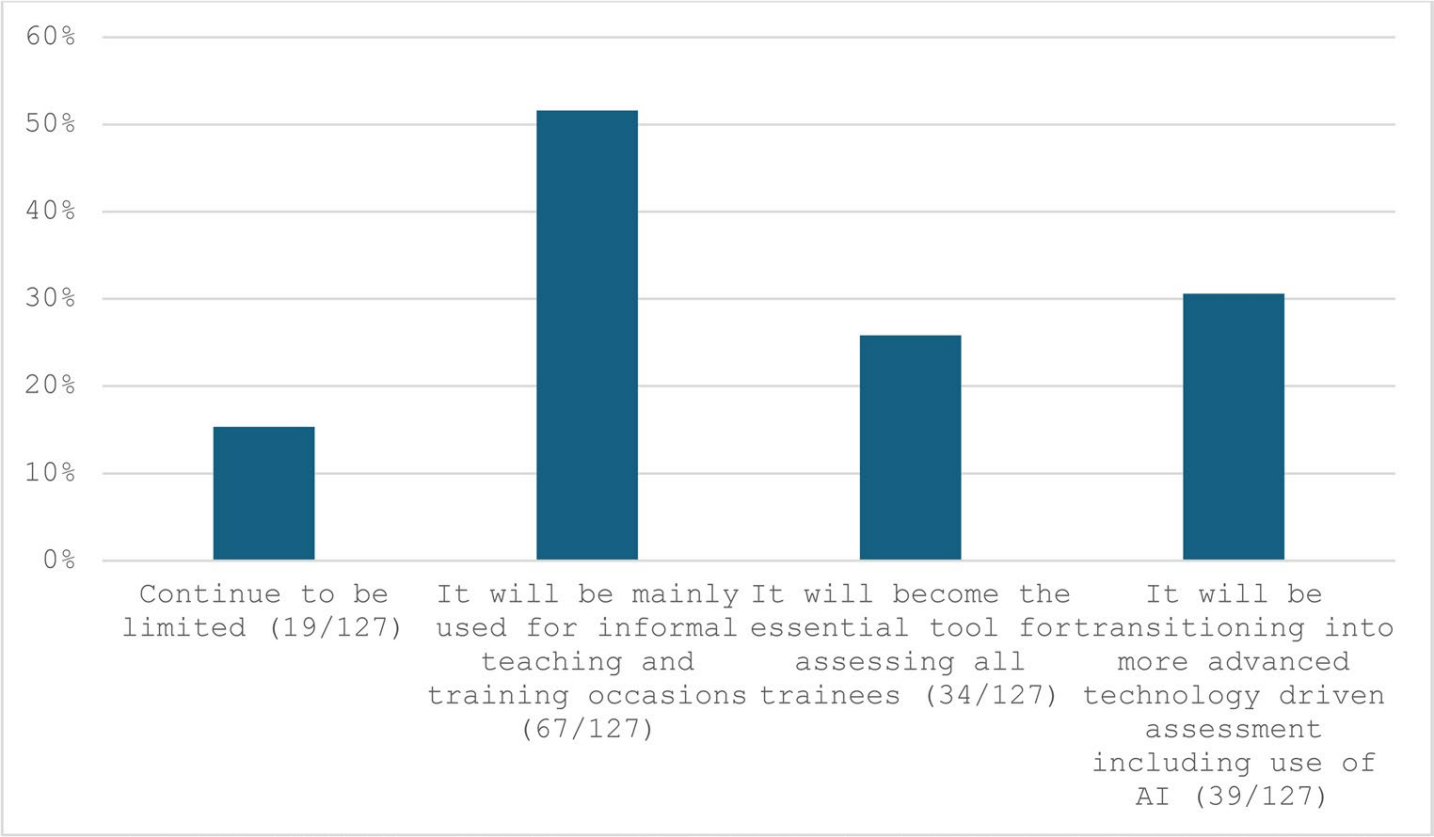
How do you envision the role of video-based assessment in the future of surgery?

The findings revealed that most surgeons (93/128, 74%) do not routinely record videos for laparoscopic surgery for review or educational purposes. When videos are recorded, the primary uses are teaching/training and self-assessment, with less frequent use for quality assurance or medicolegal purpose. (Table 1) However, a significant proportion of participants expressed that video-based assessment is “somewhat likely” or “very likely” to become a mandatory part of formal surgical training for competency evaluation. (Fig. 1) Regarding the integration of AI or software tools for video-based assessment, more participants were “somewhat supportive” (44/128, 34.4%) or “very supportive” (16/128, 12.5%) compared to those who were “unsupportive” (25/128, 19.5%) or “very against the idea” (7/128, 5.5%). (Fig. 2) Interestingly, when participants envisioned the future role of video-based assessment in surgery, only 15% (19/127) believed its use would remain limited. Conversely, 52.8% (67/127) anticipated its use for informal teaching/training, 26.8% (34/127) saw it will becoming an essential tool for all trainees, and 30.7% (39/127) suggested it would represent the future model for assessment and training. (Fig. 3)

## Discussion

This study demonstrated a consensus among general surgeons regarding the objective assessment of laparoscopic cholecystectomy, with over 75% agreement on most proposed assessment criteria. Despite not routinely recording videos, surveyed members of GSA foresee a move towards formal video-based assessment of surgical competency. The attitudes toward AI and advanced technologies in surgical assessment and training were generally positive, as indicated by this cross-sectional survey study.

The global assessment of laparoscopic surgery features key performance aspects such as depth perception, bimanual dexterity, efficiency in task execution, tissue handling and autonomy [6, 7]. The assessment items in this study aimed to translate these traditionally subjective domains into measurable criteria for laparoscopic cholecystectomy. For example, the frequency with which the gallbladder is rotated and the frequency of instrument insertion into the operative port serve as indicative metrics of the regularity with which surgeons execute specific yet prevalent tasks during laparoscopic procedures. An optimal frequency exists for a competent operator, contingent upon the complexity of the case; nevertheless, elevated frequencies are indicative of suboptimal movement economy and efficiency. In other words, an increased frequency signifies a greater incidence of superfluous and avoidable movements or actions [8]. This survey successfully gathered a broad range of expert opinions and identified potential items not included in the initial proposal. There are several limitations of this survey study regarding response rate and analysis bias. This survey focused on facilitating an examination of consensus regarding the rating criteria and viewpoints on utilizing more objective evaluative tool such as AI to enhance the purpose of assessment, the study did not perform any subgroup analyses to collect more intricate demographic inquiries, particularly in light of the potential for more unsatisfactory response rate if the survey were to exceed its three-page length. The response rate for this survey was marginally below 20%, which is lower than some published multinational survey studies in the existing literature; [9, 10] however, it is comparable to the findings reported by investigators previously engaged with specialist surgeons in the Australia and New Zealand chapter and higher than several international studies [11, 12]. This phenomenon may be ascribed to the fact that it targeted specialist surgeons with the objective of training junior surgeons, representing a distinct entity, while employing a relatively routine and commonplace procedure, which, although we consider to be significantly important and generalizable as these surgeons formed the cohort as trainers of young surgeons, this may attract comparatively less interest.

The participants largely endorsed the integration of AI into future surgical assessment and education, with many believing the surgical field is shifting towards full adoption of new technologies. This aligns with existing global AI research in video-based surgical assessment [2]. Advances in computer vision and three-dimensional neural network technology offer the potential to develop assessment items into a criterion-based scoring system and achieve automated, procedure-based assessment within surgical training [13]. The variables in this study are primarily measurable, either numerically or categorically; the resulting grading tool could be translated into a machine learning system to reduce administrative burden, provided the algorithm proves accurate and closely mirrors human-based assessment [3]. 

## Supplementary information

Below is the link to the electronic supplementary material.

Supplementary Material 1

Supplementary Material 2

Supplementary Material 3

## Data Availability

No datasets were generated or analysed during the current study.
