# Supplementary material for: An Australian perspective of using video for the assessment of laparoscopic surgery and support for artificial intelligence in performance evaluation
Source: Langenbecks Arch Surg. 2026 Mar 30;411(1):130. doi: 10.1007/s00423-026-04037-y (PMC13156208; doi:10.1007/s00423-026-04037-y)
Supplement: Supplementary file 1 — Supplementary Material 1 [file 423_2026_4037_MOESM1_ESM.pdf]

Video-based assessment of laparoscopic cholecystectomy

Dear Colleagues,

I hope this message finds you well. I am writing to invite you to participate in a survey that explores the usefulness of various items in the video-based assessment of laparoscopic cholecystectomy. Your expertise in general surgery is invaluable for developing a robust assessment tool for surgical performance.

Purpose of the Survey:

The main aim of the survey is to evaluate several items used for assessing a surgeon performing a laparoscopic cholecystectomy based on watching the video alone.

Additionally, we are keen on identifying any extra items you believe could be useful for developing an assessment tool. Your suggestions will provide valuable insights into crafting an effective evaluation method.

Time Commitment: It is designed to be short and should take only about 5 minutes of your time.

Confidentiality: Your anonymous responses will be kept strictly confidential and will only be used for research purposes. No personal identifiers will be collected.

Consent Information:

Your participation in this survey is entirely voluntary. By completing the survey, you provide consent to include your responses in our research data. If you choose not to participate, your decision will be respected and have no impact on your professional relationship with our institution. The information is collected to this survey anonymously. The findings may eventually be published, but you will not be identifiable.

If you have any questions about the survey or the consent process, please feel free to reach out, and we will be happy to provide further details.

We truly appreciate your willingness to contribute to the enhancement of surgical assessment methods. Your insights will play a critical role in shaping a more objective and comprehensive review tool, potentially improving surgical training and patient outcomes. Thank you in advance for considering this request. Your participation is greatly valued.

Best regards,

Dr Yuchen (Frank) Luo

MBBS, MSurg, PGradDipSurgAnat

General Surgery Trainee

Northern Health, Victoria

University of Melbourne PhD Research Candidate

Email: Yuchen.Frank.Luo@gmail.com

Supervisor: Dr Russell Hodgson

Email: russell.hodgson@nh.org.au

---

1) Question 1
The information collected in this survey is completely non-identifiable and used only for the purpose of research. Do you consent to proceed?

◯ Yes
◯ No

Question 2

If you are assessing a trainee's laparoscopic cholecystectomy (Exposure and dissection phase) in a videotape, how do you find the below items useful in assessment?

		Not useful	Somewhat useful	Very useful
2)	No. of times grasping Hartmann's pouch	○	○	○
3)	No. of times rotating gallbladder front to back (or vice versa) (up to achieving view of safety)	○	○	○
4)	Non-targeted diathermy	○	○	○
5)	Uncontrolled tearing of tissue	○	○	○
6)	No. times of instrument clashes	○	○	○
7)	Incorrect clipping	○	○	○
8)	Lack of progress for 1 minute	○	○	○
9)	Gallbladder perforation	○	○	○
10)	No. of times instrument inserted into the working port	○	○	○

11) Question 3
Is there any other item that you consider as also useful for assessment but not listed here? ______________________

Question 4

If you are assessing a trainee's laparoscopic cholecystectomy (Excision phase) in a videotape, which items do you consider as useful in assessment?

		Not useful	Somewhat useful	Very useful
12)	Gallbladder perforation	○	○	○
13)	Dissection into liver	○	○	○
14)	Non-targeted diathermy	○	○	○
15)	No. of times instrument inserted into the working port	○	○	○
16)	Incorrect clipping	○	○	○

17) Question 5
Is there any other item that you identify as also useful for assessment during the excision phase apart from the listed items? ______________________________

Your perspectives on video-based assessment

18) Question 6
Do you currently record laparoscopic procedures for review or educational purposes?

- ◯ Yes
- ◯ No

19) Question 7
What is your primary reason for recording the operation? (Check all that apply)

- ☐ Teaching/training
- ☐ Self-assessment for improvement
- ☐ Quality assurance
- ☐ Medicolegal purpose
- ☐ Others (Please specify)

20) If others, please specify: ______________________________

21) Question 8
How likely do you think video-based assessment of laparoscopic surgery becomes a mandatory part of formal surgical training for assessing procedural competency?

- ◯ Very unlikely
- ◯ Unlikely
- ◯ Neutral
- ◯ Somewhat likely
- ◯ Very likely

22) Question 9
How do you feel about using artificial intelligence (AI) or other software tools for video-assessment of laparoscopic surgery?

- ◯ Very against the idea
- ◯ Unsupportive
- ◯ Neutral
- ◯ Somewhat supportive
- ◯ Very supportive

23) Question 10
How do you envision the role of video-based assessment in the future of surgery?

- ☐ It will become the essential tool for assessing all trainee
- ☐ It will be mainly used for informal teaching and training occasions
- ☐ Continue to be limited
- ☐ It will be transitioning into more advanced technology driven assessment such as using AI driven systems
- ☐ Other (Please specify):

24) If others, please specify: ______________________________
