# Supplementary material for: An Australian perspective of using video for the assessment of laparoscopic surgery and support for artificial intelligence in performance evaluation
Source: Langenbecks Arch Surg. 2026 Mar 30;411(1):130. doi: 10.1007/s00423-026-04037-y (PMC13156208; doi:10.1007/s00423-026-04037-y)
Supplement: Supplementary file 2 — Supplementary Material 2 [file 423_2026_4037_MOESM2_ESM.docx]

**Invitation and consent to Participate in Video-Based Assessment of Laparoscopic Cholecystectomy Survey**

Dear Colleagues,

I hope this message finds you well. I am writing to invite you to participate in a survey that explores the usefulness of various items in the video-based assessment of laparoscopic cholecystectomy. Your expertise in general surgery is invaluable for developing a robust assessment tool for surgical performance.

**Project title:** A national survey to Australian surgical doctors regarding video-based assessment of laparoscopic cholecystectomy

**Investigator**: Dr Russell Hodgson, Dr Yuchen Luo

**Purpose of the Survey:**

The main aim of the survey is to evaluate several items used for assessing a surgeon performing a laparoscopic cholecystectomy based on watching the video alone.

Additionally, we are keen on identifying any extra items you believe could be useful for developing an assessment tool. Your suggestions will provide valuable insights into crafting an effective evaluation method.

**Time Commitment:** It is designed to be short and should take only about 5 minutes of your time.

**Confidentiality:** Your responses will be kept strictly confidential and will only be used for research purposes. No personal identifiers will be collected.

**Consent Information:**

Your participation in this survey is entirely voluntary. By completing the survey, you provide consent to include your responses in our research data. If you choose not to participate, your decision will be respected and have no impact on your professional relationship with our institution. Data collected to this survey are anonymous . The findings may eventually be published but you will not be identifiable. This project was reviewed and approved by the NH Non-HREC Ethical Review Committee.

We truly appreciate your willingness to contribute to the enhancement of surgical assessment methods. Your insights will play a critical role in shaping a more objective and comprehensive review tool, potentially improving surgical training and patient outcomes.

If you have any questions about the survey or the consent process, please feel free to reach out, and we will be happy to provide further details. Alternatively, you can also reach out to the Research Development and Governance unit (RDGU) at Northern Health.

Best regards,

Dr Yuchen (Frank) Luo, MBBS, MSurg, PhD candidate (UniMelb)

Email: [Yuchen.Luo@nh.org.au](mailto:Yuchen.Luo@nh.org.au)

Supervisor: Dr Russell Hodgson, MBBS, PhD, FRACS

Email: [Russell.Hodgson@nh.org.au](mailto:Russell.Hodgson@nh.org.au)

Research Development and Governance Unit (RDGU), Northern Health

Telephone: (03) 8405 2918

Email: [ResearchDGU@nh.org.au](mailto:ResearchDGU@nh.org.au)
