# Supplementary material for: An Australian perspective of using video for the assessment of laparoscopic surgery and support for artificial intelligence in performance evaluation
Source: Langenbecks Arch Surg. 2026 Mar 30;411(1):130. doi: 10.1007/s00423-026-04037-y (PMC13156208; doi:10.1007/s00423-026-04037-y)
Supplement: Supplementary file 3 — Supplementary Material 3 [file 423_2026_4037_MOESM3_ESM.docx]

**Supplementary table 1 – CHERRIES checklist**

| Checklist for Reporting Results of Internet E-Surveys (CHERRIES) | Description |
| --- | --- |
| Ethics approval | Ethics was approved by the Northern Health Research Development and Governance through the NH non-HREC pathway. Reference number 2025_Non-HREC_10  Study was then submitted to General Surgeons Australia (GSA) through the GSA Research & Scholarships Committee for second approval for distribution of survey to active GSA members. |
| Informed consent | Survey was pre-designed and made available through REDcap website using the Northern Health research login details. A comprehensive introductory email explained the design of this study, purpose of the survey and outcomes anticipated from participants. Question one on the survey was a question of consent for participation itself. |
| Data protection | Survey was published using REDcap account owned by Northern Health department of research, and the data was deidentified as there was no mechanism for recording identifiable data. Data was then transferred to a spreadsheet database and securely stored on Northern Health servers by the primary investigator. |
| Development and pre-testing | Survey assessment items were based on previous panel discussion, literature review, pilot study investigating interrater reliability as mentioned in manuscript. |
| Recruitment process | The survey was published using REDcap and distributed to the members of GSA via email with the support of GSA research board. We allowed for 30 days for response time and collected results directly from REDcap website and transferred the data to a spreadsheet database stored in Northern Health. |
| Survey administration | It was a voluntary survey with informed consent as its question one. There were no incentives offered. To prevent biases, the survey did not collect any subcategorized demographics or any identifiable information. The items were initially proposed and then reviewed in departmental research meetings to restrict to a three-page survey and maximum 10 questions to aide in response rate. |
| Response rates | 20.0% as expected based on investigator’s previous engagement with surveys to GSA members. |
| Preventing multiple entries from the same participant | REDcap requires a link through invitation emails distributed. Although the link was the same, each individual was provided with a data entry number on REDcap immediately as they answered question one (informed consent), and they were allowed to re-entre or complete the questions should they wish to return to the survey. |
| Analysis | No advanced statistical analysis was required. All percentage calculation and reports were generated automatically by REDcap. |
